# Tracking Lower Urinary Tract Symptoms and Tamsulosin Side Effects Among Older Men Using a Mobile App (PERSONAL): Feasibility and Usability Study

**DOI:** 10.2196/30762

**Published:** 2021-12-10

**Authors:** Austin W Lee, Stacey A Kenfield, Elizabeth Y Wang, Anthony Enriquez, Akinyemi Oni-Orisan, Michael A Steinman, Ida Sim, Benjamin N Breyer, Scott R Bauer

**Affiliations:** 1 Department of Urology University of California, San Francisco San Francisco, CA United States; 2 Department of Epidemiology and Biostatistics University of California, San Francisco San Francisco, CA United States; 3 Columbia University Vagelos College of Physicians and Surgeons New York City, CA United States; 4 Department of Clinical Pharmacy and Institute for Human Genetics University of California, San Francisco San Francisco, CA United States; 5 Veterans Affairs Medical Center University of California, San Francisco San Francisco, CA United States; 6 Division of Geriatrics Department of Medicine University of California, San Francisco San Francisco, CA United States; 7 Division of General Internal Medicine Department of Medicine University of California, San Francisco San Francisco, CA United States

**Keywords:** LUTS, tamsulosin, mobile, app, mobile phone

## Abstract

**Background:**

Continuous α1a-blockade is the first-line treatment for lower urinary tract symptoms (LUTS) among older men with suspected benign prostatic hyperplasia. Variable efficacy and safety for individual men necessitate a more personalized, data-driven approach to prescribing and deprescribing tamsulosin for LUTS in older men.

**Objective:**

We aim to evaluate the feasibility and usability of the PERSONAL (Placebo–Controlled, Randomized, Patient-Selected Outcomes, N-of-1 Trials) mobile app for tracking daily LUTS severity and medication side effects among older men receiving chronic tamsulosin therapy.

**Methods:**

We recruited patients from the University of California, San Francisco health care system to participate in a 2-week pilot study. The primary objectives were to assess recruitment feasibility, study completion rates, frequency of symptom tracking, duration of tracking sessions, and app usability rankings measured using a follow-up survey. As secondary outcomes, we evaluated whether daily symptom tracking led to changes in LUTS severity, perceptions of tamsulosin, overall quality of life, medication adherence between baseline and follow-up surveys, and perceived app utility.

**Results:**

We enrolled 19 men within 23 days, and 100% (19/19) of the participants completed the study. Each participant selected a unique combination of symptoms to track and recorded data in the PERSONAL app, with a median daily completion rate of 79% (11/14 days). The median duration of the app session was 44 (IQR 33) seconds. On a scale of 1 (strongly disagree) to 5 (strongly agree), the participants reported that the PERSONAL app was easy to use (mean 4.3, SD 1.0), that others could learn to use it quickly (mean 4.2, SD 0.9), and that they felt confident using the app (mean 4.4, SD 0.8). LUTS severity, quality of life, and medication adherence remained unchanged after the 2-week study period. Fewer men were satisfied with tamsulosin after using the app (14/19, 74% vs 17/19, 89% at baseline), although the perceived benefit from tamsulosin remained unchanged (18/19, 95% at baseline and at follow-up). In total, 58% (11/19) of the participants agreed that the PERSONAL app could help people like them manage their urinary symptoms.

**Conclusions:**

This pilot study demonstrated the high feasibility and usability of the PERSONAL mobile app to track patient-selected urinary symptoms and medication side effects among older men taking tamsulosin to manage LUTS. We observed that daily symptom monitoring had no adverse effects on the secondary outcomes. This proof-of-concept study establishes a framework for future mobile app studies, such as digital n-of-1 trials, to collect comprehensive individual-level data for personalized LUTS management in older men.

## Introduction

### Background

Lower urinary tract symptoms (LUTS) affect more than half of men over the age of 70 years [1] and are associated with an increased risk of falls [2] and psychological distress that impairs health-related quality of life [3]. Clinical practice guidelines for the management of LUTS as a result of presumed benign prostatic hyperplasia list α_1a_-blockers, such as tamsulosin, as the first-line therapy [4]. Widespread adoption of α_1a_-blocker therapy for prolonged durations may expose patients, particularly those who are nonresponders, to unnecessary potential harms such as orthostatic hypotension and dizziness, which can result in falls [5] and polypharmacy [6].

Several considerations challenge the existing practice of continuous α_1a_-blocker use in older men with LUTS. First, randomized clinical trials demonstrate modest efficacy of tamsulosin and other α_1a_-blockers for improving LUTS, as well as a large placebo effect [7]. This limits the ability of clinicians to identify true responders to chronic tamsulosin therapy and the degree of symptomatic improvement attributable to tamsulosin alone [7,8]. In fact, several small studies have indicated no symptomatic worsening after tamsulosin discontinuation among men with chronic LUTS [9-11] or men concurrently taking a 5α-reductase inhibitor [12-15]. The landmark trials that informed societal guidelines were also conducted predominantly among healthy and younger (age <65 years) White men [7] and may not be generalizable to more racially diverse and older men with multimorbidity and polypharmacy who are most likely to be receiving chronic tamsulosin therapy [16,17]. Fewer benefits and greater harm in this population have led to recommendations against the use of α_1a_-blockers in older men [18,19]. However, tamsulosin may be safe and effective for a subset of older men with LUTS. A more personalized approach to identifying which older men will and will not benefit from continuing long-term tamsulosin therapy is urgently needed.

Mobile health (mHealth) apps are an emerging platform for personalized health care delivery that have thus far been underused in benign urology [20]. The ability of mHealth apps to achieve high-throughput, low-burden data collection [21] on a wide range of patient-centric outcomes could facilitate daily tracking of LUTS severity and side effects among men receiving chronic LUTS therapy. Previous efforts using mobile apps to track patient-reported outcomes have informed patient-driven care and helped patients with heart failure and depression identify and self-manage potential symptom triggers [22,23]. If men are able and willing to track their symptoms while undergoing LUTS treatment (eg, formal n-of-1 trials or self-experimentation), their data can be used to generate individualized estimates of benefits and harms of both prescribed and self-management LUTS treatments [24-30]. To reduce the burden of frequent symptom assessments, we designed the PERSONAL (Placebo–Controlled, Randomized, Patient-Selected Outcomes, N-of-1 Trials) mHealth app for collecting daily LUTS severity and medication side effect data. However, whether older men are able and willing to use a mobile app to track their LUTS and medication side effects, particularly while receiving chronic α_1a_-blocker therapy, represents a knowledge gap.

### Objectives

In this paper, we report the results of a 2-week pilot study using the PERSONAL mobile app to monitor daily LUTS severity and medication side effects among older men receiving chronic tamsulosin therapy. Our primary objectives are to assess the feasibility and usability of the PERSONAL app as demonstrated by time to reach full recruitment, study completion rates, frequency of symptom tracking, duration of tracking sessions, and app usability rankings. As secondary objectives, we explore whether quality of life measures, medication adherence, LUTS severity, and perception of tamsulosin changed after using the app, as well as the perceived utility of the app. We hypothesize that daily use of the PERSONAL app would not significantly impact these secondary outcomes.

## Methods

### Design and Setting

We recruited patients from the University of California, San Francisco health care system to participate in a 2-week mobile app–based study to evaluate the feasibility and usability of the PERSONAL app for tracking LUTS severity and tamsulosin side effects. This study was approved by the University of California, San Francisco Institutional Review Board (institutional review board approval: 19-28557**)**, and all participants provided informed consent.

### Recruitment

**Figure 1:**
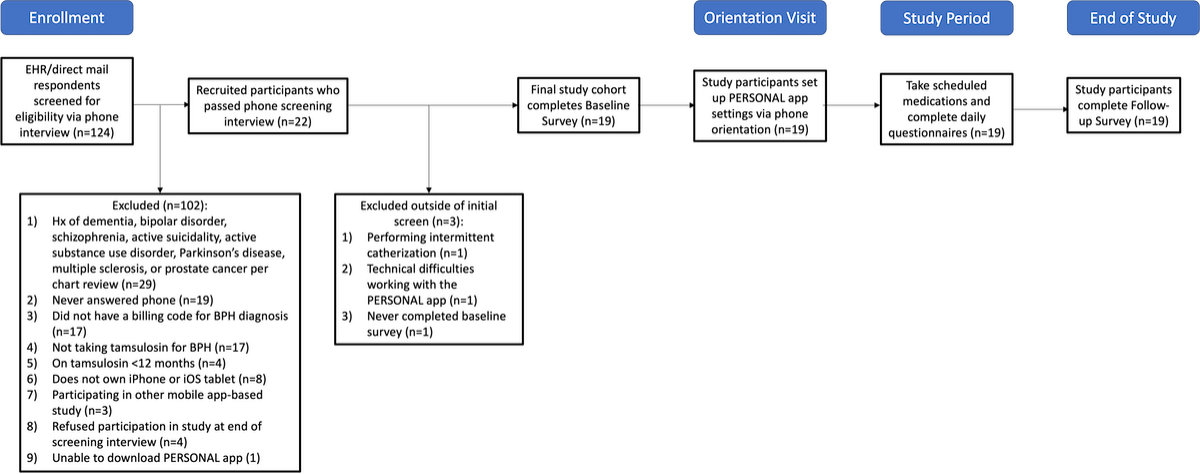
Study flow diagram. BPH: benign prostatic hyperplasia; EHR: electronic health record; Hx: history; PERSONAL: Placebo–Controlled, Randomized, Patient-Selected Outcomes, N-of-1 Trials.

We invited eligible male patients aged >55 years who were monitored by urology clinicians from the University of California, San Francisco, who had previously agreed to be contacted by researchers via the electronic medical record, and who had a diagnosis of LUTS or benign prostatic hyperplasia on the basis of International Classification of Diseases-10 billing codes (N40.x) and an active prescription for tamsulosin hydrochloride to participate in this study (Figure 1). On the basis of prior mHealth studies, we determined that we would have 90% power to observe at least one failed primary outcome during this feasibility study with a target sample size of 20 participants. Eligible patients received an invitation by secure message via the electronic medical record portal describing the PERSONAL app and study, and those who were not enrolled in secure messaging received a mailed letter with the same information. Patients who expressed interest in participating were screened for inclusion and exclusion criteria by telephone (Multimedia Appendix 1). Eligible patients were then sent a baseline questionnaire using REDCap (Research Electronic Data Capture; Vanderbilt University), instructed on how to download the PERSONAL app (available for free on the iOS App Store at the time of the study), and scheduled for a phone orientation visit with a study team member.

### Orientation and Pilot

**Figure 2:**
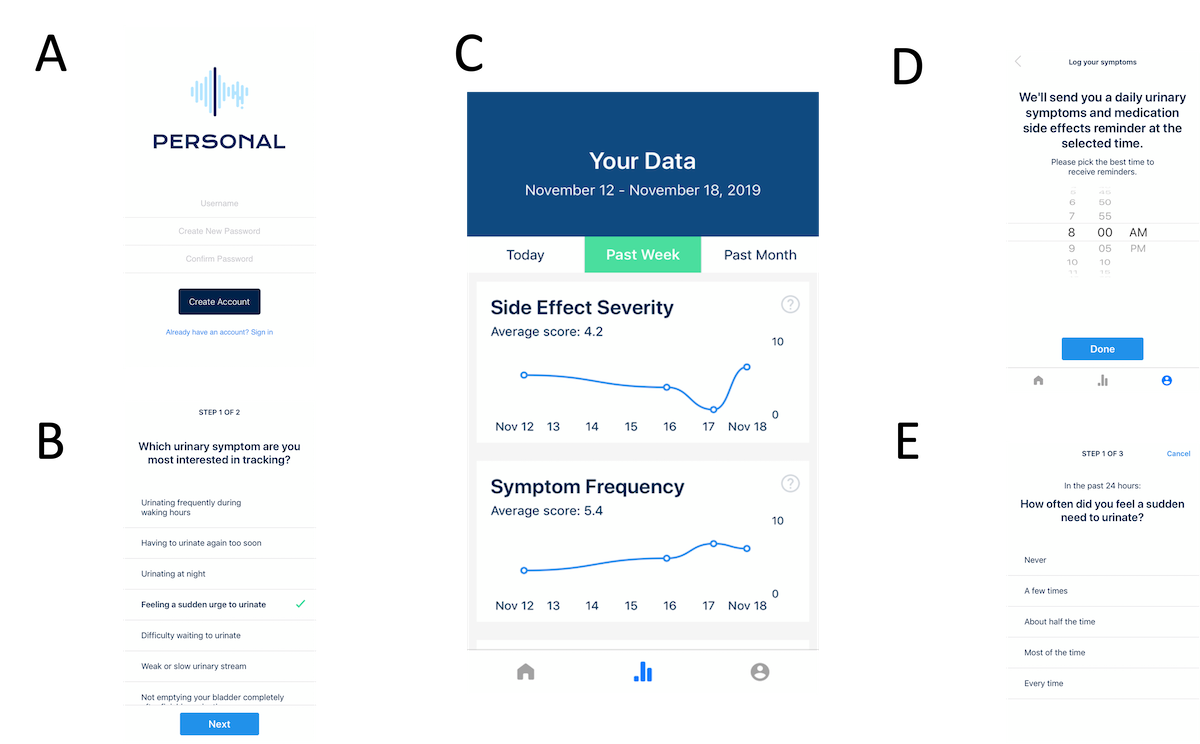
Representative screenshots of the PERSONAL app interface. (A) Login. (B) Urinary symptom selection. (C) Personalized graphs of changes in symptom and side effect severity over time. (D) Daily app reminder setup. (E) Daily symptom and side effect severity questionnaire. PERSONAL: Placebo–Controlled, Randomized, Patient-Selected Outcomes, N-of-1 Trials.

The study subjects participated in their orientation visit via a phone call with a study team member who confirmed or facilitated the installation of the PERSONAL app on the participant’s iOS device. Following verbal cues from the study team member, the participants then customized the app (Figure 2A) by selecting at least 1 urinary symptom from a list of 10 and at least 1 tamsulosin side effect from a list of 12, sourced from the tamsulosin Food and Drug Administration label, to track daily for 2 weeks. The participants were informed that this list included potential tamsulosin side effects but that they might also experience them because of reasons unrelated to tamsulosin. Patient-selected symptoms and side effects were used instead of a general symptom questionnaire to minimize the daily time spent answering questions on the PERSONAL app and to maximize the utility of the app for each participant by focusing on the symptoms they found most bothersome (Figures 2B and 2D). The participants were recommended to track 2 to 3 urinary symptoms and 2 to 3 potential medication side effects, but they were allowed to track as many symptoms and side effects as they desired (Multimedia Appendix 2). They also selected the time they preferred for receiving app reminder notifications. After completing the app setup, the participants were oriented on the rest of the PERSONAL app features and practiced recording their first symptoms and side effects. At the end of the orientation visit, the participants were provided with study team contact information for technical app support as needed throughout the study period.

At baseline, we collected participant demographics, social and health-related behaviors, and medical history through a REDCap survey. Physical activity, alcohol use, and social connection or isolation were assessed using the Institute of Medicine Measures of Social and Behavioral Determinants of Health and categorized according to established thresholds [31]. We also assessed the perceptions of the participants regarding medication burden and appropriateness, their willingness to stop medications, and their desire to be involved in medication decisions via the revised Patients’ Attitudes Toward Deprescribing (rPATD) questionnaire (not specific to tamsulosin) [32].

The participants used the PERSONAL app for 2 weeks and completed daily symptom and side effect questionnaires to track the urinary symptoms and potential tamsulosin side effects they had selected (Figure 2E). At any point during the study, the participants were able to graphically visualize their symptom and side effect severity scores over time within the PERSONAL app (Figure 2C). The total number and duration of the tracking sessions were recorded using the PERSONAL app (Multimedia Appendix 3).

### Primary Outcomes

To evaluate feasibility, we assessed the time elapsed until our recruitment goal was reached, the percentage of participants who completed the study, the mean number of days with completed symptom tracking, and the mean duration of the tracking sessions. To evaluate usability, the participants completed a previously published and validated usability scale [33] via REDCap asking them to rate qualitative statements describing the app’s frequency of use, ease of use, integration of functions, and ability to be learned by new users, as well as confidence using the app. We also assessed the experience of the patients with the phone-based PERSONAL orientation visit and app setup to determine if in-person orientation visits are needed for future studies. We set the following benchmarks to measure study and app effectiveness: (1) recruitment and retention of 20 eligible men would be completed within 3 to 6 months, (2) >70% of participants would complete the study, (3) the daily questionnaire completion rate would be >50%, (4) the average symptom logging session duration would be <2 minutes, and (5) the mean rating of ease of use of the app would be >3 on a scale of 1 (strongly disagree) to 5 (strongly agree).

### Secondary Outcomes

Secondary outcomes were assessed using baseline and follow-up surveys. These included LUTS severity, perceptions of tamsulosin, overall quality of life, and medication adherence. The perceived utility of the app was also assessed at the end of the study.

LUTS severity was measured using questions selected from the validated Lower Urinary Tract Dysfunction Research Network Comprehensive Assessment of Self-Reported Urinary Symptoms questionnaire item bank, which was generated following the principles of the Patient-Reported Outcomes Measurement Information System (PROMIS) initiative funded by the National Institutes of Health [34]. Specifically, 8 questions were asked to assess urinary urgency (frequency of urge episodes and severity of urgency), daytime frequency (number of voids and interval between voids), nocturia, slow or weak urine flow, incomplete emptying, and postvoid dribbling. The responses to these questions were added to obtain a composite LUTS severity score (range 0-30). An additional 8 questions were asked to assess urinary incontinence subtypes, including urgency, stress, and unspecified urinary incontinence. The responses were added to obtain a composite urinary incontinence severity score (range 0-32). The perceptions of tamsulosin were evaluated using questions that inquired whether the participants were satisfied with tamsulosin and whether they felt any perceived benefits from it.

We evaluated the health-related quality of life of the patients using the PROMIS-29 v2.0 [35]. PROMIS scores represent standardized T-scores for a given domain referenced to a population with mean 50 and SD 10, where a higher PROMIS T-score represents having more of the given domain (ie, a higher PROMIS T-score for anxiety represents an individual having greater anxiety). PROMIS T-score thresholds can be interpreted as follows for symptoms (ie, anxiety, depression, fatigue, sleep disturbance, and pain interference): 0 to 55, within normal limits; >55 to 60, mild; >60 to 70, moderate; and >70, severe. PROMIS T-score thresholds can be interpreted as follows for domains (ie, physical function and ability to participate in social roles): >45, within normal limits; >40 to 45, mild deficit; >30 to 40, moderate deficit; ≤30, severe deficit.

Medication adherence was measured using the Voils Medication Adherence score [36]. The Voils Medication Adherence score ranges from 1 to 5, with higher scores indicating a greater extent of medication nonadherence. Finally, we evaluated the perceptions of the patients regarding the utility of the PERSONAL app on the basis of standardized questionnaires from previous mHealth studies [28].

Mean and SD were calculated for scores with normal distribution, and median and IQR were calculated for scores with skewed distribution. Statistical significance was set at *P*<.05. All analyses were performed using STATA (version 15.1; StataCorp LLC).

## Results

### Demographics

After our initial study invitation, 124 men expressed interest in the study, 102 were excluded via telephone screening, and 19 were ultimately enrolled in the study between March 3 and March 26, 2020 (Figure 1). The mean age of the participants was 70 (SD 7) years, and 32% (6/19) of them self-identified their race or ethnicity as Asian, Hispanic, or other (Table 1). Most participants had completed a bachelor’s degree or higher (16/19, 84%) and reported no difficulty in paying basic living expenses (14/19, 74%). More than a third of the participants were socially isolated (7/19, 37% on the basis of frequency of communication or getting together with family, friends, or neighbors in a typical week; frequency of attending religious services, clubs, or organizations in the past year; and marital status), and 21% (4/19) screened positive for alcohol use. Hypertension, prostatitis, and visual impairment were the most common comorbidities, with a mean of 2 (SD 1.5) comorbidities per participant. The participants generally disagreed that medications were burdensome (mean rPATD score 2.7, SD 1.0), that their current medication regimen was inappropriate (mean rPATD score 2.7, SD 0.8), and that they had a willingness to stop medications (mean rPATD score 2.2, SD 0.8), and agreed that they desired to be involved in their medication regimen determination (mean rPATD score 4.2, SD 0.5).

**Table 1:** Demographics, health-related behaviors, and medical history of 19 study participants.

Demographic					Value
Age (years), mean (SD)					70 (7)
**Race or ethnicity, n (%)**					
	White, non-Hispanic			13 (68)	
	Black			0 (0)	
	Asian			3 (16)	
	Hispanic			2 (11)	
	Other			1 (5)	
**Highest degree earned, n (%)**					
	High school diploma			2 (11)	
	Associate degree			1 (5)	
	Bachelor’s degree			5 (26)	
	Master’s degree			8 (42)	
	Doctorate or professional			3 (16)	
**Ability to pay for basic living expenses, n (%)**					
	Not hard at all			14 (74)	
	Somewhat hard			4 (21)	
	Very hard			0 (0)	
	Prefer not to answer			1 (5)	
**Social and health-related behaviors, n (%)**					
	**Physical activity^a^**				
		Inactive	3 (16)		
		Insufficiently active	3 (16)		
		Sufficiently active	13 (68)		
	Ever smoked at least 100 cigarettes			11 (58)	
	Current smoking			0 (0)	
	Positive screen for alcohol use^b^			4 (21)	
	**Social connection or isolation score^c^**				
		Most isolated	7 (37)		
		Very isolated	1 (5)		
		Somewhat isolated	8 (42)		
		Not isolated	3 (16)		
**Self-reported medical history**					
	**Comorbidities, n (%)**				
		Hypertension	10 (53)		
		Coronary artery disease	5 (26)		
		Angina	2 (11)		
		Congestive heart failure	1 (5)		
		Chronic obstructive pulmonary disease	0 (0)		
		Diabetes	2 (11)		
		Stroke or intracerebral hemorrhage	2 (11)		
		Parkinson disease or multiple sclerosis	0 (0)		
		Visual impairment	6 (32)		
		Prostatitis	9 (47)		
	Number of comorbidities, mean (SD)			2 (1.5)	
**Revised Patients’ Attitudes Toward Deprescribing score, mean (SD)**					
	Burden subscore			2.7 (1.0)	
	Appropriateness subscore			2.7 (0.8)	
	Willingness to stop medications subscore			2.2 (0.8)	
	Involvement in medications subscore			4.2 (0.5)	

^a^Physical activity was calculated in minutes per week engaged in moderate to strenuous activity and was categorized as *inactive* (0 min/week), *insufficiently active* (1–149 min/week), and *sufficiently active* (150+ min/week).

^b^Alcohol use was tabulated as a composite value integrating alcohol consumption frequency (*How often do you have a drink?*) and density (*How many standard drinks on a typical day? How often do you have ≥6 drinks on one occasion?*), and a score of ≥4 indicated a positive screening.

^c^Social isolation was assessed as a composite value integrating not interacting with others, not attending social gatherings (church, meetings, or clubs), and not being married. The values could then be interpreted as follows: *most isolated* (0 to 1 point), *very isolated* (2 points), *somewhat isolated* (3 points), and *not isolated* (4 points).

### Feasibility

**Figure 3:**
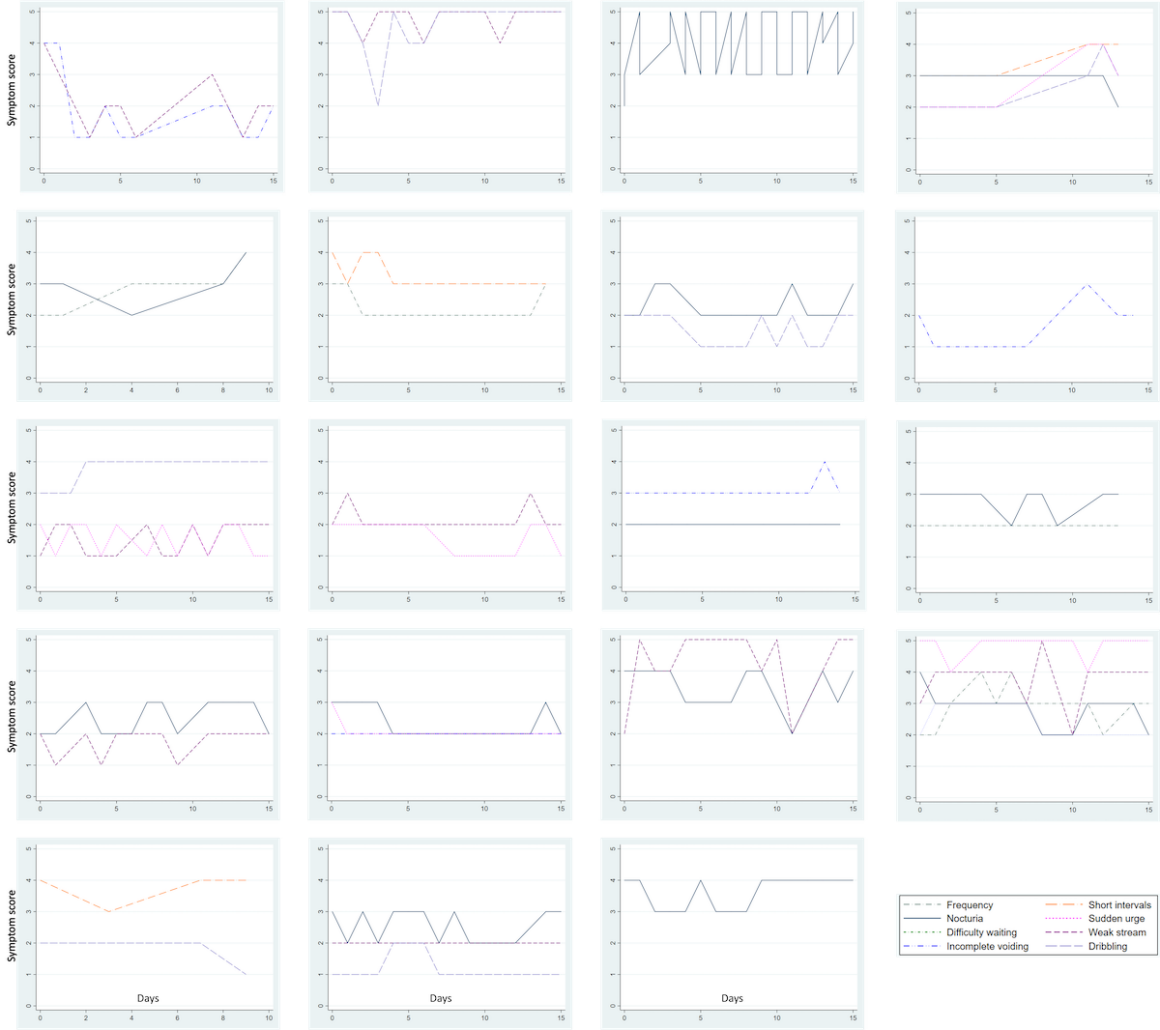
Change in urinary symptom severity over time for 19 study participants. Each panel in this figure visualizes the change in urinary symptom severity for an individual study participant.

The time required to reach our enrollment goal was 23 days. A total of 95% (19/20) of the enrolled participants completed the study (1 participant was enrolled but did not complete the baseline survey and was therefore excluded from subsequent analyses) and tracked their symptoms on the PERSONAL app for a median of 11 out of 14 (IQR 3) days. They spent a median of 44 (IQR 33) seconds on each tracking session. When symptom severity scores were visualized over time, we observed significant heterogeneity among the participants, with some men exhibiting highly variable symptom severity and others reporting stable symptom severity (Figure 3).

### Usability

The PERSONAL app usability results are presented in Table 2. On a scale of 1 (strongly disagree) to 5 (strongly agree), the participants generally agreed that the PERSONAL app was easy to use (mean 4.3, SD 1.0), that they could imagine people learning to use it quickly (mean 4.2, SD 0.9), and that they felt confident using the app (mean 4.4, SD 0.8). They agreed less on whether they would use the app frequently (mean 2.7, SD 1.2). The participants generally disagreed with statements describing the PERSONAL app as awkward to use, having a high learning curve, or requiring technical support to use. They agreed that the phone-based orientation was useful.

**Table 2:** Perceived usability of PERSONAL (Placebo–Controlled, Randomized, Patient-Selected Outcomes, N-of-1 Trials) app.

Experience^a^			Value, mean (SD)
**PERSONAL app**			
	**Pros**		
		Would use app frequently	2.7 (1.2)
		Easy to use	4.3 (1.0)
		Various functions well integrated	3.5 (1.2)
		Imagine people would learn to use quickly	4.2 (0.9)
		Felt confident using app	4.4 (0.8)
	**Cons**		
		Found app unnecessarily complex	1.7 (0.9)
		Would need technical support to use	1.5 (0.9)
		Too much inconsistency in app	1.9 (0.8)
		Awkward to use	1.6 (0.9)
		High learning curve	1.5 (0.9)
**PERSONAL orientation visit and app setup**			
	**Pros**		
		Comprehensive to get started with study	4.3 (0.8)
		Tracked symptoms most important to patient	4.2 (0.7)
	**Cons**		
		Could set up app without orientation	3.6 (1.3)
		In-person orientation would be more helpful	1.5 (0.8)
		Needed more guidance after	1.7 (1.0)
		Unable to track all prioritized symptoms	2.7 (1.1)
		Unable to track all prioritized side effects	2.1 (1.0)

^a^Scale: 1, *strongly disagree,* to 5, *strongly agree*.

### Secondary Outcomes

Table 3 displays the results of the secondary outcomes assessed at baseline and follow-up. LUTS severity remained unchanged throughout the study period (LUTS severity score at baseline: mean 12, SD 5; at follow-up: mean 12, SD 5). After using the PERSONAL app for 2 weeks, fewer men were satisfied with tamsulosin (14/19, 89% of the participants responded *Yes* when asked “*Taking all things into account, are you satisfied with your tamsulosin medication?*” vs 17/19, 74% at baseline), although the perceived benefits from tamsulosin remained unchanged (18/19, 95% responded *Yes* when asked “*Have you had any benefit from your tamsulosin medication?*” at both baseline and follow-up). Overall, the participants reported normal health-related quality of life at baseline, and their PROMIS T-scores did not worsen after using the PERSONAL app for 2 weeks. The participants also reported high medication adherence before and after using the PERSONAL app (Voils score at baseline: mean 2.3, SD 0.2; at follow-up: mean 2.4, SD 0.3).

**Table 3:** Patient-reported secondary outcomes at baseline and follow-up.

Secondary outcome			Baseline	Follow-up
**LUTS^a^ severity and treatment satisfaction**				
	LUTS severity score^b^, mean (SD)		12 (5)	12 (5)
	Urinary incontinence severity score^c^, mean (SD)		1 (2)	1 (1)
	Extremely or very bothered by urinary symptoms, n (%)		3 (16)	2 (11)
	Satisfied with tamsulosin, n (%)		17 (89)	14 (74)
	Any perceived benefit from tamsulosin, n (%)		18 (95)	18 (95)
**Quality of life**				
	**PROMIS^d^-29 v2.0 T-scores, mean (SD)**			
		Physical function	52 (8)	52 (8)
		Anxiety	52 (10)	51 (9)
		Depression	48 (8)	46 (7)
		Fatigue	47 (10)	47 (9)
		Sleep disturbance	56 (5)	54 (4)
		Ability to participate in social roles	56 (10)	52 (10)
		Pain interference	50 (11)	50 (9)
**Medication adherence**				
	Voils Medication Adherence score, mean (SD)		2.3 (0.2)	2.4 (0.3)

^a^LUTS: lower urinary tract symptoms.

^b^Sum of nonincontinence items from the Lower Urinary Tract Dysfunction Research Network assessing urgency, daytime frequency, nocturia, slow or weak urine flow, incomplete emptying, and postvoid dribbling (range 0-30; higher score indicates greater severity).

^c^Sum of incontinence items from the Lower Urinary Tract Dysfunction Research Network assessing urgency, stress, and unspecified urinary incontinence (range 0-32; higher score indicates greater severity).

^d^PROMIS: Patient-Reported Outcomes Measurement Information System.

### Utility

The perceived utility of the PERSONAL app is presented in Table 4. More than half (11/19, 58%) of the participants reported that the PERSONAL app could help people like them manage their urinary symptoms. In the absence of additional guidance or clinician involvement, the participants rated the utility of the PERSONAL app on a scale of 1 (not at all helpful) to 5 (extremely helpful) for keeping track of symptoms (mean 2.9, SD 1.6), working with physicians to achieve treatment goals (mean 3.0, SD 1.6), noticing things that help with urinary symptoms (mean 2.6, SD 1.6), and building confidence in the approach to managing urinary symptoms (mean 2.9, SD 1.7).

**Table 4:** Perceived utility of PERSONAL (Placebo–Controlled, Randomized, Patient-Selected Outcomes, N-of-1 Trials) app.

Item			Value
Belief that PERSONAL app can help manage LUTS^a^, n (%)			11 (58)
**Perceived utility of PERSONAL app for LUTS management^b^, mean (SD)**			
	Keeping track of symptoms	2.9 (1.6)	
	Working with physicians to achieve treatment goals	3.0 (1.6)	
	Identifying urinary symptom triggers	2.3 (1.5)	
	Noticing things that help with urinary symptoms	2.6 (1.6)	
	Confidence in approach to urinary symptoms	2.9 (1.7)	

^a^LUTS: lower urinary tract symptoms.

^b^Scale: 1, *not at all helpful* to 5, *extremely helpful*.

## Discussion

### Principal Findings

In this pilot study of a mobile app for tracking daily LUTS severity and medication side effects, we demonstrated that recruiting and retaining older men receiving chronic tamsulosin therapy to use the PERSONAL app is highly feasible; the participants were able to complete the study and track their symptoms almost every day for 2 weeks, and more than half of them found the app helpful despite no clinician involvement or additional guidance beyond symptom tracking. We also observed no adverse effects of daily symptom monitoring on the secondary outcomes of LUTS severity, health-related quality of life, and medication adherence. Interestingly, fewer participants reported satisfaction with tamsulosin after the symptom tracking period.

### Feasibility of Tracking LUTS With the PERSONAL App

As a proof-of-concept study, the PERSONAL pilot demonstrated that a mobile app could be used to track daily symptoms and side effects among older men taking tamsulosin for LUTS. The participants agreed that the app was easy to use, that people could learn to use it quickly, and that they felt confident using the app without additional guidance. High observed rates of daily questionnaire completion suggest that the interface of the app and questionnaire length were not so burdensome as to discourage the participants from tracking their symptoms. The duration of the average tracking session further showed how quickly men could record their daily symptoms. The findings that the PERSONAL app can build high user engagement in a feasible length of time align with those of similar studies assessing mobile app feasibility in optimizing psychotherapy in community psychiatric clinics and routine follow-up care in breast cancer survivors [37,38]. Although longer follow-up is needed to determine how the duration of tracking influences sustained engagement and perceived burden, these findings indicate that it is possible to collect a large volume of data on urinary symptoms and medication side effects from older men with relatively low perceived burden for a period of at least 2 weeks. High-volume individualized data may be particularly valuable in clinical or research settings where changes (or lack thereof) in symptoms or side effects observed after an intervention or modification of treatment strategy could inform the patient and their clinician and guide subsequent management decisions, although this hypothesis needs to be tested in future studies.

### Heterogeneity in Patient-Selected Symptom Tracking

Of note was the degree of heterogeneity in both the symptoms prioritized for tracking among the participants and daily symptom severity during the pilot. LUTS can refer to a range of different symptoms, including both voiding sequelae such as hesitancy, poor stream, straining, prolonged urination, incomplete bladder emptying, and dribbling, and storage sequelae such as frequency, urgency incontinence, and nocturia [39]. Consequently, follow-up studies have identified different urinary symptoms as the most bothersome, among them urge urinary incontinence [40,41] and nocturia [42]. The reasons for this heterogeneity include underlying anatomical pathophysiology, personal lifestyle, cultural backgrounds, and psychosocial factors. Regardless, the capacity to capture such heterogeneity at the individual level allows for the directed management of the particular symptoms of a patient rather than a broader diagnosis. Similarly, variability in symptom severity hinges on factors such as cultural mindsets toward health and a subjective threshold to report symptoms [43], further emphasizing the need for more granular data and individualized recommendations.

### Monitoring for Unintended Consequences of Daily Symptom Tracking

After tracking their symptoms for 2 weeks using the PERSONAL app, the participants did not experience changes in LUTS severity, quality of life, or medication adherence. This may be because such perceptions of LUTS, quality of life, and medication adherence are more fixed opinions rather than perspectives that easily fluctuate, especially during a short-term study. It is also possible that, because this was a single-arm pilot without a comparator, the participants were not able to experience what life off tamsulosin might be like—which could have subsequent implications for the perception of LUTS, quality of life, and medication adherence. Although short-term studies with daily symptom tracking appear unlikely to have unintended adverse consequences, the risk may increase with studies of longer duration. Despite no change in perceptions of adherence, fewer participants were satisfied with tamsulosin after using the PERSONAL app for 2 weeks. One potential reason for this finding is that tracking their symptoms and side effects showed the participants direct evidence that their most bothersome symptoms were poorly controlled or that they experienced side effects more often than they expected. Increased monitoring may have also made them aware that their symptoms did not necessarily correlate with the dosing schedule of tamsulosin, thus diminishing its benefit. The app could also have served as a physical reminder of the burden of taking daily medication, resulting in less satisfaction with tamsulosin itself. Although our sample size was small for this pilot study, these findings serve as testable hypotheses for further exploration.

### Perceived Utility of the PERSONAL App

More than half of the participants agreed that the PERSONAL app would be helpful when working with a clinician to achieve their treatment goals. They believed that these additional data on the day-to-day variability in LUTS severity and medication side effects might influence LUTS management and could be useful to share with their clinician. However, in the absence of more detailed guidance or clinician involvement, the participants found the PERSONAL app neither helpful nor unhelpful. This finding suggests that men do require additional support to interpret the variability in their symptoms and connect this variability to various symptom triggers or treatments. In the absence of self-experimentation or alternating treatments, the PERSONAL app does not help participants determine if either their self-management techniques or tamsulosin is effective in reducing LUTS severity or side effects. This information will be used to develop more personalized recommendations for participants in larger planned individualized crossover (or n-of-1) trials using the PERSONAL app.

### Strengths and Limitations

The strengths of our study include the demonstration of successful remote patient recruitment and enrollment, high patient engagement and participation, and the novel use of patient-selected outcomes to minimize participant burden and maximize the number of repeated symptom assessments. This study also provides key feasibility and usability data to guide improvements in the PERSONAL app. This low-burden approach to data collection using the PERSONAL app may also facilitate the use of individualized crossover or n-of-1 trials [44] to further personalize LUTS management by testing interventions with short wash-out periods, such as tamsulosin therapy, on an individual patient. Subsequent integration of self-experimentation or n-of-1 trial protocols into a mobile app would allow patients to assess their personalized response to specific therapies in terms of LUTS severity and intervention side effects [45].

Our study has some limitations. First, although one-third of the study participants were people of color, we did not recruit any Black or African American participants and cannot comment on whether the PERSONAL app has similar feasibility or usability outcomes in this population. Future studies will require more explicit recruitment goals and customized approaches to recruit a more diverse patient population, including transcultural adaptations of questionnaires and surveys. Second, the participants in our pilot study were highly educated and had a high socioeconomic status, although 21% (4/19) found it somewhat difficult to pay for basic living expenses. This limits our ability to comment on whether other populations of older men who may not be as educated or wealthy may have greater difficulty using an mHealth app to track their symptoms and medication side effects. However, other studies have demonstrated widespread accessibility of and comfort with smartphone use irrespective of socioeconomic status [46]. Third, our pilot study used detailed baseline and follow-up questionnaires that may have impacted the desire of the participants to enroll in the study. In future studies, it may be possible to reduce the number of question items while still capturing appropriate granularity, particularly for questionnaires that participants will complete repeatedly during study follow-up. Fourth, we used a version of the rPATD that assesses general attitudes toward deprescribing rather than a more recently developed version that is specific to tamsulosin [47]. However, we found that men in both studies reported similar attitudes regarding willingness to consider targeted deprescribing. Finally, the participants remained on the same LUTS treatment throughout the study period; therefore, we were unable to provide them with personalized estimates of tamsulosin efficacy or side effects, which is a direction for future study.

### Conclusions

In conclusion, the PERSONAL app pilot demonstrated high feasibility and usability of a mobile app for tracking and visualizing daily LUTS severity and medication side effects among older men receiving chronic tamsulosin therapy. The PERSONAL app did not change the LUTS severity, health-related quality of life, or medication adherence of the patients. Future directions of study include leveraging the high-volume data collected via the PERSONAL app to develop individualized estimates of benefits and harms from LUTS interventions in older men.

## Appendix

Multimedia Appendix 1 Study inclusion and exclusion criteria.

Multimedia Appendix 2 Participants’ ranking of most bothersome lower urinary tract symptoms and tamsulosin side effects to track using the PERSONAL (Placebo–Controlled, Randomized, Patient-Selected Outcomes, N-of-1 Trials) app.

Multimedia Appendix 3 Scale for daily urinary symptom scoring.

## Abbreviations

LUTS: lower urinary tract symptoms
mHealth: mobile health
PERSONAL: Placebo–Controlled, Randomized, Patient-Selected Outcomes, N-of-1 Trials
PROMIS: Patient-Reported Outcomes Measurement Information System
REDCap: Research Electronic Data Capture
rPATD: revised Patients’ Attitudes Toward Deprescribing
