# Supplementary material for: Tracking Lower Urinary Tract Symptoms and Tamsulosin Side Effects Among Older Men Using a Mobile App (PERSONAL): Feasibility and Usability Study
Source: JMIR Form Res. 2021 Dec 10;5(12):e30762. doi: 10.2196/30762 (PMC8709917; doi:10.2196/30762)
Supplement: Multimedia Appendix 1 [file formative_v5i12e30762_app1.doc]

Supplemental Table 1. Study inclusion and exclusion criteria.

| **Inclusion Criteria** |
| --- |
| 1. Male patient evaluated by a urologist at the University of California, San Francisco 2. iPhone smartphone or iOS tablet with an active data plan and/or connected to a home WiFi network 3. Age ≥55 years 4. Diagnosis code for BPH or other micturition problem based on ICD-10 (ICD10 N40, R35.0-1, R39.11-12, R39-15-16, R39198) 5. Taking tamsulosin daily for ≥12 months 6. Downloaded an app from the App Store within the past year 7. Ability to speak and read in English 8. Willing to install the PERSONAL mobile application on their iOS device |
| **Exclusion Criteria** |
| 1. History of acute urinary retention, recurrent urinary tract infections, nephrolithiasis, obstructive kidney disease, urethral stent, or intermittent catherization 2. Active cancer treatment or medical condition that would limit the patient’s life expectancy to <6 months per chart review 3. History of dementia, bipolar disorder, schizophrenia, active suicidality, active substance use disorder, Parkinson’s disease, multiple sclerosis, or prostate cancer per chart review 4. Current participation in another mobile health app-based clinical study 5. Planning to relocate from study area within 6 months 6. Impaired vision that limits the use of mobile health apps 7. Have a health proxy |

BPH: benign prostatic hyperplasia; ICD: international classification of diseases
