# Supplementary material for: Tracking Lower Urinary Tract Symptoms and Tamsulosin Side Effects Among Older Men Using a Mobile App (PERSONAL): Feasibility and Usability Study
Source: JMIR Form Res. 2021 Dec 10;5(12):e30762. doi: 10.2196/30762 (PMC8709917; doi:10.2196/30762)
Supplement: Multimedia Appendix 2 [file formative_v5i12e30762_app2.doc]

Supplemental Table 2. Participants’ ranking of most bothersome lower urinary tract symptoms and tamsulosin side effects to track using the PERSONAL app.

|  | **#1 Most Bothersome** | **#2 Most Bothersome** | **#3 Most Bothersome** |
| --- | --- | --- | --- |
| Lower Urinary Tract Symptoms, n (%) |  |  |  |
| Waking up and urinating frequently during the night | 10 (53) | 0 (0) | 1 (5) |
| Bladder not completely empty after urination | 2 (11) | 4 (21) | 2 (11) |
| Feeling a sudden urge to urinate | 2 (11) | 1 (5) | 2 (11) |
| Slow or weak urine flow | 1 (5) | 4 (21) | 7 (37) |
| Dribbling urine just after zipping pants or pulling up underwear | 1 (5) | 3 (16) | 5 (26) |
| Difficult to wait more than a few minutes once you notice the urge to urinate | 1 (5) | 3 (16) | 0 (0) |
| Urinating frequently while awake | 1 (5) | 2 (11) | 2 (11) |
| Short period of time between urinations | 1 (5) | 1 (5) | 0 (0) |
|  |  |  |  |
| Potential Tamsulosin Side Effects, n (%) |  |  |  |
| Erection or ejaculation difficulties | 7 (37) | 0 (0) | 2 (11) |
| Back pain | 2 (11) | 3 (16) | 3 (16) |
| Fatigue or excessive tiredness | 2 (11) | 1 (5) | 1 (5) |
| Runny nose | 2 (11) | 0 (0) | 1 (5) |
| Insomnia or difficulty sleeping | 1 (5) | 2 (11) | 0 (0) |
| Constipation | 1 (5) | 1 (5) | 1 (5) |
| Dizziness or lightheadedness | 1 (5) | 0 (0) | 1 (5) |
| Decreased libido or sex drive | 0 (0) | 3 (16) | 1 (5) |
| Weakness | 0 (0) | 2 (11) | 1 (5) |
| Headache | 0 (0) | 1 (5) | 0 (0) |
| Diarrhea | 0 (0) | 1 (5) | 0 (0) |
| Nausea | 0 (0) | 0 (0) | 2 (11) |
