# Supplementary material for: Tracking Lower Urinary Tract Symptoms and Tamsulosin Side Effects Among Older Men Using a Mobile App (PERSONAL): Feasibility and Usability Study
Source: JMIR Form Res. 2021 Dec 10;5(12):e30762. doi: 10.2196/30762 (PMC8709917; doi:10.2196/30762)
Supplement: Multimedia Appendix 3 [file formative_v5i12e30762_app3.doc]

Supplemental Table 3. Scale for Daily Urinary Symptom Scoring.

| Question | Scale |
| --- | --- |
| In the past 7 days, during waking hours, how many times did you typically urinate? | 0: 3 or fewer times (a day)  1: 4-7 times (a day)  2: 8-10 times (a day)  3: 11 or more times (a day) |
| In the past 7 days, during a typical day, how much time typically passed between urinations? | 0: More than 6 hours  1: 5-6 hours  2: 3-4 hours  3: 1-2 hours  4: Less than 1 hour |
| In the past 7 days, during a typical night, how many times did you wake up and urinate? | 0: none  1: 1 time  2: 2-3 times  3: More than 3 times |
| In the past 7 days, how often did you feel a sudden need to urinate? | 0: Never  1: A few times  2: About half the time  3: Most of the time  4: Every time |
| In the past 7 days, once you noticed the need to urinate, how difficult was it to wait more than a few minutes? | 0: Not difficult  1: A little difficult  2: Somewhat difficult  3: Very difficult  4: Unable to wait |
| In the past 7 days, how often was your urine flow slow or weak? | 0: Never  1: A few times  2: About half the time  3: Most of the time  4: Every time |
| In the past 7 days, how often did you feel that your bladder was not completely empty after urination? | 0: Never  1: A few times  2: About half the time  3: Most of the time  4: Every time |
| In the past 7 days, how often did you dribble urine just after zipping your pants or pulling up your underwear? | 0: Never  1: A few times  2: About half the time  3: Most of the time  4: Every time |
| In the past 7 days, how often did you completely lose control of your bladder? | 0: Never  1: A few times  2: About half the time  3: Most of the time  4: Every time |
| In the past 7 days, how often did you leak or wet a pad after feeling a sudden need to urinate? | 0: Never  1: A few times  2: About half the time  3: Most of the time  4: Every time |
| In the past 7 days, how often did you leak urine or wet a pad while laughing, sneezing, or coughing? | 0: Never  1: A few times  2: About half the time  3: Most of the time  4: Every time |
| In the past 7 days, how often did you leak urine or wet a pad when doing physical activities, such as exercising or lifting a heavy object? | 0: Never  1: A few times  2: About half the time  3: Most of the time  4: Every time |
